# The impact of opioid analgesics with concomitant antipsychotic use on pain modulation and management in internal medicine: a cross-sequential study protocol

**DOI:** 10.3389/fpain.2025.1500422

**Published:** 2025-04-08

**Authors:** Nicola Grignoli, Simone Livoti, Angela Greco, Michela Pironi, Roberta Noseda, Alessandro Ceschi, Maria Luisa Garo, Luca Gabutti

**Affiliations:** ^1^ Cantonal Sociopsychiatric Organisation, Public Health Division, Department of Health and Social Care, Repubblica e Cantone Ticino, Switzerland; ^2^ Department of Internal Medicine, Ente Ospedaliero Cantonale, Bellinzona, Switzerland; ^3^ Faculty of Biomedical Sciences, Family Medicine Institute, Università della Svizzera Italiana, Lugano, Switzerland; ^4^ Quality and Patient Safety Service, Ente Ospedaliero Cantonale, Locarno, Switzerland; ^5^ Faculty of Economics, University of Tor Vergata, Rome, Italy; ^6^ Division of Clinical Pharmacology and Toxicology, Institute of Pharmacological Sciences of Southern Switzerland, Ente Ospedaliero Cantonale, Lugano, Switzerland; ^7^ Faculty of Biomedical Sciences, Università della Svizzera Italiana, Lugano, Switzerland; ^8^ Clinical Trial Unit, Ente Ospedaliero Cantonale, Lugano, Switzerland; ^9^ Department of Clinical Pharmacology and Toxicology, University Hospital Zurich, Zurich, Switzerland; ^10^ Biostatistics Unit, Mathsly Research, Rome, Italy

**Keywords:** pain, mental health, opioids, antipsychotics, internal medicine, hospitalization

## Abstract

**Background:**

Acute and chronic pain represents an escalating public health concern, necessitating safer and more effective in-hospital management approaches, including mental health. New treatment combinations involving psycholeptics are rising, but real-world evidence is lacking.

**Objectives:**

The study's primary objective is to evaluate the impact of combined opioid analgesics and antipsychotics in-hospital medication on pain modulation. The secondary objective is to evaluate pain management.

**Methods:**

The cross-sequential study designed by this protocol will analyze retrospective data on 5,000 hospital admissions over four years (2019–2023) gathered from Electronic Health Records (EHR) of a multisite hospital in southern Switzerland. Eligible patients are aged 18 or older and hospitalized in an Internal Medicine ward. All patients with documented pain intensity assessment through a Visual Analogue Scale (VAS ≥ 1) will be included. Cross-sectional data on demographic and clinical variables and type of medication (opioid analgesics, antipsychotics, and selected other drugs according to the Anatomical Therapeutic Chemical classification system) will be screened at hospital admission (T1) and discharge (T2). Pain modulation will be assessed by gravity (VAS mean), intensity (VAS peak/extreme value), and pain treatment effectiveness (*Δ*T2-T1 VAS). Hospitalization paths (short- and long-term readmissions and total length of hospital stays) will be scrutinized as additional longitudinal indices for pain management and excluded from the cross-sectional analysis. A mixed model approach will assess VAS changes from T1 to T2. Logistic regression and regression models for count data will be used for short- and long-term readmission, respectively. Propensity score matching will be used to mitigate selection bias.

**Discussion:**

This methodological approach combines cross-sectional and longitudinal EHR data gathering in a cross-sequential design. This integration allows for a comprehensive examination of pain modulation and management among internal medicine recipients of concomitant opioids and antipsychotic treatment, spanning both hospitalization and post-discharge periods. By leveraging EHR data, the study protocol ensures reliability and standardization while minimizing missing information. Additionally, the protocol addresses the potential limitations of observational designs.

**Conclusions:**

This method offers a comprehensive and rigorous approach to investigating pain modulation and management in internal medicine patients receiving combined opioid analgesics and antipsychotics, with potential implications for enhancing clinical practice and healthcare resource utilization.

## 1 Introduction

Pain is an underestimated and undertreated issue in internal medicine departments (1, 2). In-hospital pain management quality is still challenged by several factors, such as adverse effects of analgesics, lack of specific medical training, communication issues, and misperception of pain itself (3, 4). Internal medicine physicians deal with complex drug prescription paths and need specific clinical and patient-centered information guidance. Concerns are mainly raised about opioid prescribing for chronic pain conditions and the challenge of managing long-term treatment within primary care (5, 6). It is particularly crucial to weigh treatment alternatives to reduce the risk of long-term misuse or addiction (7, 8). Moreover, unresolved pain is increasingly recognised as influencing internal medicine hospital readmissions. Data show that pain at admission increases the risk of unplanned hospital returns (9), pain at discharge predicts early readmissions (10), and effective in-hospital pain treatment prevents further hospitalisation in high-utilizer patients (11). However, integrated approaches focused on the multidimensionality of pain are lacking and need to be tested for clinical significance (12, 13).

The current understanding of pain requires explicitly targeting its emotional and cognitive dimensions in treatment options (14, 15). Solid evidence has indeed established that anxiety, depression, and stress can modulate pain perception and contribute to the chronicity of pain conditions (16–18). Several clinical-embedded screening options exist that can tackle the psychological dimension of chronic pain (18, 19); among them, catastrophizing is one of the most impactful factors, and research is starting to consider it to optimise treatment decision-making (20). Psychopharmacological research is growing in this field. The direct and indirect antalgic effect mediated by some antidepressants by the activation of some adrenergic and serotonergic pathways is well-established in the literature (21–23). The potential antalgic role of psycholeptic drugs is increasingly considered, particularly for chronic pain. Growing evidence suggests that antipsychotics can effectively target the emotional aspects of pain when integrated into currently available pain treatments (24, 25). Specific neural antalgic pathways investing dopamine are currently being studied (26, 27), but such psychopharmacology progress has not yet been tested in clinical practice. Some studies have shown promising evidence, particularly for olanzapine in central sensitization, fibromyalgia and migraine (28). The only Cochrane review currently available on acute and chronic pain (29) emphasizes that despite the potential antalgic effect, their use has so far been limited by possible sedative and extrapyramidal adverse effects for older-generation antipsychotics. Integrating antipsychotics into pain treatment can indeed lead to complex pharmacodynamic and pharmacokinetic interactions that may increase the risk of side effects of antalgics, such as sedation, cognitive impairment, and respiratory depression. Second- and third-generation antipsychotics have better tolerability profiles and less extrapyramidal effects than their predecessors (30), though caution is needed regarding potential weight gain, metabolic issues, and overdose risk with sedative agents.

“Real-world” data on the combination of opioid analgesics with antipsychotics in pain treatment are needed as a fast way to gain insight into clinical settings and inspire future clinical studies (31). This protocol describes a retrospective study on internal medicine electronic health records (EHR) that allows information gathering on pain modulation (i.e., pain gravity, intensity, and effectiveness in pain reduction) and management (clinical indices and hospitalization paths). The objective is to determine how the concomitant use of opioid analgesics and antipsychotics impacts pain modulation and management compared to the use of opioid analgesics alone.

## 2 Methods

### 2.1 Study design and data gathering

This protocol designs a cross-sequential study (32, 33). This methodological design allows for a comprehensive examination of pain modulation and management among internal medicine recipients of opioid analgesics and antipsychotic treatments, spanning hospitalization and post-discharge periods. The group of patients receiving opioid analgesics and antipsychotics will be compared with the control group of patients treated with opioid analgesics alone. The study was designed to improve the presentation of data conforming to STROBE Statement (34, 35).

**Figure 1:**
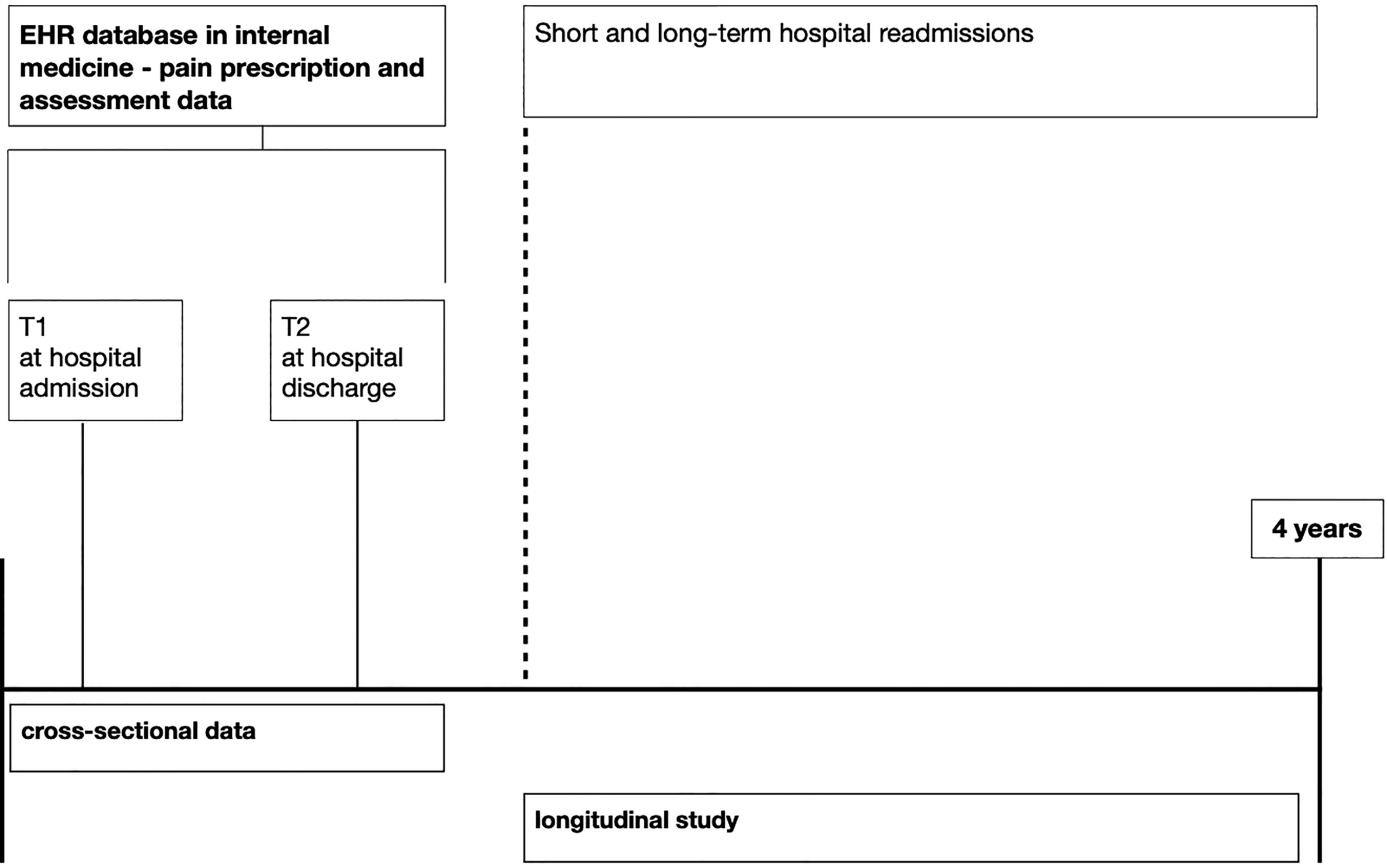
Timeline for data gathering.

Data will be retrospectively gathered through EHR over four years (2019–2023) from the Internal Medicine Department of the Ente Ospedaliero Cantonale, a network of public hospitals in Switzerland. EHRs are hospital-quality data that generate standardized and reliable health-related information on hospital functioning. Linkages between different subsets of the database are made using a unique number assigned to each episode of care. For this project, EHR data will be extracted anonymously and aggregated by the Ente Ospedaliero Cantonale Information Technology Department. No information about the person's name or address will be extracted. The dataset will, therefore, be completely anonymized. The quality of information will be measured for each record by a variable derived from the mean of three criteria (i.e., accuracy, completeness, and timeliness), each rated on a 5-point scale, with 1 indicating very low quality and 5 indicating very high quality. Follow-up data will be gathered concerning short-term readmission and re-hospitalization in the long term. See Figure 1 for an illustration of the protocol design and the timeline for data gathering.

### 2.2 Sample selection

Eligible patients will be 18 years or older and admitted to the Ente Ospedaliero Cantonale Internal Medicine Department, which comprises four centrally managed and coordinated hospital sites across southern Switzerland. All patients with documented pain intensity assessment through a Visual Analogue Scale (VAS ≥ 1) (36) will be included. All referrals to hospitalization are considered for the study. Admission for patients younger than 18 years, with a less than 24 h length of hospital stay or documented contraindications to the drugs of interest in the present study, will be excluded.

Hospitalized patients screened for the study are financially covered by the Swiss Lamal insurance system. The internal medicine ward physicians carry out the pharmacological treatment. In Ente Ospedaliero Cantonale hospitals, a VAS rating on a 10-point scale is a mandatory nursing assessment for pain. Pain checks with VAS are performed at admission and every nurse shift during hospitalisation; the last measurement will be considered for the analysis on discharge. If the pain requires specific interventions (i.e., surgery), nursing pain surveillance is activated after VAS assessment, and its activation will be screened for the study. For patients unable to rate their pain on a VAS scale, a Pain Aid scale is employed, and these patients are excluded from the study.

### 2.3 Sample size

Ente Ospedaliero Cantonale data show that the Internal Medicine Department received around 50,000 admissions in 4 years: 30% of the admitted patients have at-admission benzodiazepines or Z-Drugs medication and 11.5% have at-admission antipsychotic medication (37). Internal quality data show that in the Internal Medicine Department, 10% of the admitted patients have at-admission opioid analgesics. We expect, therefore, to screen retrospectively 5,000 admissions for patients with antipsychotic medication.

The sample size required for this study is 1,952 (opioids and antipsychotics group: 488, opioids alone group: 1,464), value determined using the Wilcoxon-Mann–Whitney test with the following parameters:

– Variable of interest: mean VAS recorded during the entire hospitalization;
– Effect size: 0.15, a value hypothesized by the authors based on their clinical experience;
– Alpha: 0.05;
– Power: 0.80;
– Allocation ratio N2/N1: 3.

The G*Power software was used to determine the sample size.

### 2.4 Measurements

Table 1 describes cross-sectional and longitudinal data to be obtained from EHR on hospital admissions to the Ente Ospedaliero Cantonale Internal Medicine Department between the 1st of October 2019 and the 30th of September 2023. Information on demographical data, hospital and clinical data, pain modulation, pain treatment paths, pain management clinical indices, and pain management hospitalization paths will be screened. The complete list of variables retrospectively collected is reported in Table 1.

**Table 1:** Cross-sectional and longitudinal data obtained from HER classified per type, factor and EHR source.

Cross sectional data		
Type of data	Factor	EHR source
Demographical	Age	Number of years
	Sex	Female or Male
Hospital and clinical	Hospital site	Name of the Hospital
	Nursing pain surveillance activation	Yes or No
	Type of admission	Elective or Urgent
	Severity of illness score	Case Mix on disease severity, i.e., mean cost weight
Pain modulation	Pain gravity	Hospitalization VAS mean
	Pain intensity	Hospitalization VAS peak
	Pain treatment effectiveness	*Δ*VAS from admission to discharge (last assessment)
Pain treatment paths	Type and number of opioid analgesics prescriptions	Name of the molecule
		Number of molecules
	Type and number of antipsychotics prescriptions	Name of the molecule
		Number of molecules
Pain management clinical Indices	Length of hospital stay	Number of days
	Risk of in-hospital fall	Yes or No
	Risk of delirium	Yes or No
	Type of discharge	Home, Hospital Transfer, Rehabilitation, Psychiatric clinic
Longitudinal data		
Variable	Factor	EHR Data
Pain management hospitalization paths	Short-term readmission (within 1 month)	Yes or No
	Long-term readmission (until 9 months)	Yes or No
	Frequency of readmission	Number of Hospital admissions
	Long-term hospitalizations	Total length of hospital stays in days

Data on opioid analgesics and antipsychotic prescriptions will be captured at two endpoints: T1 prescription at the hospital admission and T2 therapy at discharge; the molecule's type of the drugs will be screened without considering the dosage. The two separate endpoints will allow the capture of chronic pain treatments by the screening of prior medication at admission with new in-hospital medication still prescribed at discharge. Additional data on in-hospital pain-related medication will be gathered from EHR accounting for acute pain treatment.

Opioids, antipsychotics and a broad list of molecules will be screened to evaluate complex pharmacological interactions on pain neural pathways, including other psycholeptics, psychoanalytic, antiepileptics and selected other drugs according to the Anatomical Therapeutic Chemical classification system (38). Principal and secondary molecules screened in the study are defined in Table 2. In the analysis, we will make a qualitative further distinction for the type of opioid analgesics between strong (morphine, methadone, fentanyl, oxycodone, buprenorphine, tapentadol, hydromorphone, oxymorphone) and weak (codeine, dihydrocodeine and tramadol) (39) and for type of antipsychotics between first-generation (such as haloperidol and flupenthixol), second-generation (such as olanzapine and quetiapine) and third-generation (aripiprazole, brexpiprazole and cariprazine) (40).

**Table 2:** List of screened molecules with potential impact on pain modulation/pain neural pathway prescriptible in Switzerland categorized according to ATC classification.

Anatomical therapeutic chemical classification system (ATC) category	Subtype of chemical classification
Main molecules	
N02A opioids	N02AA Natural opium alkaloids; N02AB Phenylpiperidine derivatives; N02AE Oripavine derivatives; N02AF Morphine derivatives; N02AJ Opioids with non-opioid analgesics; N02AX Other opioids
N05A antipsychotics, neuroleptics	N05AA Phenothiazines with aliphatic side chain; N05AD Butyrophenone derivatives; N05AE Indole derivatives; N05AF Thioxanthene derivatives; N05AH Diazepines, oxazepines, thiazepines and ossepines; N05AL Benzamides; N05AN Lithium; N05AX Other antipsychotics
Secondary molecules	
N02 analgesics	**N02B Other analgesics and antipyretics**
	N02BA Salicylic acid and derivatives; N02BB Pyrazolone; N02BE Anilides; N02BF Gabapentinoids; N02BG Other analgesics and antipyretics
	**NO2C Anti-migraine preparations**
	N02CC Selective serotonin (5HT1) agonists; N02CD Calcitonin gene-related peptide (CGRP) antagonists; N02CX Other antimigraine preparations
N03A antiepileptics	N03AA Barbiturates and derivatives; N03AB Hydantoin derivatives; N03AD Succinimide derivatives; N03AE Benzodiazepine derivatives; N03AF Carboxamide derivatives; N03AG Fatty acid derivatives; N03AX Other antiepileptics
N05 psycholeptics	**N05B Anxiolytics**
	N05BA Benzodiazepine derivatives; N05BB Diphenylmethane derivatives
	**N05C Hypnotics and sedatives**
	N05CC Aldehydes and derivatives; N05CD Benzodiazepine derivatives; N05CF Benzodiazepine related drugs; N05CH Melatonin receptor agonists; N05CM Other hypnotics and sedatives; N05CX Hypnotics and sedatives in combination, excluding barbiturates
N06 psychoanaleptics	**N06A Antidepressants**
	N06AA non-selective monoamine reuptake inhibitors; N06AB Selective serotonin reuptake inhibitors; N06AG Monoamine oxidase inhibitors A; N06AX Other antidepressants
	**N06B Psychostimulants, agents used for ADHD and nootropics**
	N06BA Centrally acting sympathomimetics; N06BC Xanthine derivatives; N06BX Other psychostimulants and nootropics
	**N06C Psycholeptics and psychoanaleptics in combination**
	N06CA Antidepressants in combination with psycholeptics
N07 other nervous system drugs	**N07B Drug used in addictive disorders**
	N07BA Drugs used in nicotine dependence; N07BB Drugs used in alcohol dependence; N07BC Drugs used in opioid dependence

Pain management clinical indices rely on the risk of in-hospital falls, and the risk of delirium, evaluated during the nurses' admission assessment through standardised questions and categorised as binary variables. Length of hospital stay and type of discharge will also be screened. Longitudinal data on hospitalization paths (short- and long-term readmissions, long-term hospitalization) will also be scrutinized within the same database, allowing further pain management categorization (see Table 1). In the case of repeated admissions, only the data from the first hospitalization will be considered. Ente Ospedaliero Cantonale EHR database does not allow the screening of psychiatric diagnoses, pain-related diagnoses, drug dosage, side effects, or adverse events related to drug prescription, and this data will not be screened.

## 3 Outcomes

Pain modulation measured as pain gravity (mean VAS), pain intensity (VAS peak/extreme value), and pain treatment effectiveness (*Δ*T2-T1 VAS) will be three separate primary outcomes. Subsequently, VAS will be assessed by providing a score categorized as follows: VAS ≤ 3 (i.e., mild pain), VAS 3-7 (i.e., moderate pain), and VAS >7 (i.e., severe pain). This categorization aligns with established guidelines for interpreting VAS scores among patients with chronic musculoskeletal pain (41). VAS at admission and discharge (last assessment) covariates will be studied (see Table 1). Short- and long-term readmissions will be secondary outcomes.

### 3.1 Research hypothesis

This is the main research hypothesis to be tested. Null Hypothesis (H0): The concomitant use of opioid analgesics and antipsychotics has no impact on the effectiveness of pain modulation and management compared to the use of opioid analgesics alone. Alternative Hypothesis (Ha): The concomitant use of opioid analgesics and antipsychotics significantly improves pain modulation and management compared to the use of opioid analgesics alone.

## 4 Data analysis

### 4.1 Main analysis

Quantitative variables will be presented as mean and standard deviation or as medians with 25th and 75th percentiles after checking variables distribution through the Kolmogorov–Smirnov test. Counts and percentages will be used to describe categorical and dichotomous variables. Baseline characteristics of the study population will be compared between patients treated with opioid analgesics plus antipsychotics and patients treated with opioid analgesics alone using the Pearson *χ*^2^ test for categorical variables and the Wilcoxon rank sum test for continuous variables if not normally distributed; otherwise comparisons on continuous variables between the two groups will be performed through *t*-test.

Propensity score matching will be used to consider the differences between the two groups at T1. Before propensity score matching, the demographic and clinical characteristics at baseline will be compared using the Standardized Mean Difference. Propensity scores will be estimated using a logistic regression model that considers patient-specific demographic characteristics (age and gender) and clinical characteristics at baseline (VAS severity, severity of illness, previous home treatment with opioids and/or antipsychotics). The predictors will be selected based on expert opinion. Statistical interference will also examine other possible predictors related to specific characteristics of the sample. Subsequently, patients treated with opioids and antipsychotics (intervention group) will be compared in a 1:1 ratio without replacement with patients treated with opioids alone (control group), using a propensity score with a caliper of 0.2 of the standard deviation of the logit of the propensity score, as suggested by Austin (42). Standardized Mean Differences will also be calculated in the matched sample to compare baseline characteristics between the two groups. A Standardized Mean Difference <0.10 will be used to indicate adequate matching (43, 44). Changes in VAS score from T1 and T2 will be analyzed using a mixed-linear approach (fixed effect: time, group, interaction term time*group, interaction term time*concurrent pharmacological treatments; random effects: Patients). Subgroup analyses will also be performed to evaluate the impact of the type and the number of antipsychotics and/or opioids. The appropriateness of the mixed model will be assessed by testing linearity, normality of residuals, homogeneous error variance, autocorrelation of errors, independence of residuals, and variances of random effects. Since the short-term readmission variable (hospital readmission within 1 month of discharge) is dichotomous, univariate and multivariate logistic regression analyses will be performed to assess the probability of short-term readmission in patients treated with opioids and antipsychotics or opioids alone. Poisson regression or zero-inflated negative binomial regression will be used for the number of hospital readmissions. The choice between these two approaches will be made by analyzing the distribution of the number of hospital readmissions. Finally, the relationship between the type of treatment (opioids and antipsychotics vs. opioids alone) and the average length of stay will be assessed by means of a multiple Ordinary Least Squares regression analysis.

### 4.2 Ancillary analysis

In addition to the primary analysis comparing patients discharged on opioid therapy alone with patients receiving opioids in combination with antipsychotics, an ancillary analysis will be conducted in patients who received these treatments only during hospitalization without continuing them at discharge. This subgroup consists of patients with acute pain who need to be treated in hospital but do not require long-term therapy. The aim of this analysis is to describe the clinical characteristics of these patients, assess short-term outcomes such as pain control and compare these findings with those of the primary analysis. The same statistical methods will be used as in the primary analysis. This ancillary analysis will provide further insight into the role of short-term use of opioids and antipsychotics in hospitalized patients.

### 4.3 Sensitivity analysis

After assessing the type and quantity of missing values, a sensitive analysis will be also performed using different approaches to eliminate possible biases due to these values. A sensitive analysis will be also carried out with regard to the quality of the information collected. For all analyses, two-tailed *p*-values less than 0.05 will be considered significant. Statistical analyses will be performed using STATA18 (StataCorp., College Station, TX, USA).

## 5 Ethics and dissemination

The study protocol is outside the Swiss Federal Human Research Act (HRA, RS 810.30: https://www.fedlex.admin.ch/eli/cc/2013/617/en#a51), a clarification of responsibility confirming the legal frame for the study has been requested and released by the regional Ethics committee (Comitato etico cantonale, Repubblica Cantone Ticino, Req-2024-00282). Patient consent is not required for this study. Health-related data will be extracted, analyzed, and presented in aggregated form, granting full data anonymization. There will be neither patient nor public involvement in the project.

## 6 Anticipated results

This protocol designs a cross-sequential study combining the effectiveness of a retrospective cross-sectional study with the opportunity to describe developmental change offered by a longitudinal study (32, 45). In particular, the adopted methodology will gather data on pain modulation and management during and after hospitalization, gaining potential insight into pain chronicization prevention and healthcare resource use (re-hospitalization). The study protocol requires a large sample size defined with a rigorous method of data selection, gathering the adequate statistical power and reproducibility needed to be conclusive (46). EHR data will combine highly reliable and standardized medical and nurses' hospital quality data: each entry for each health professional group is mandatory for in-hospital admission and discharge. In addition, evaluation with VAS is automatically requested by the patient's health record once the pain is experienced by a patient. The combination of such data aims to collect real-world data that is highly reproducible and promises to gain translational evidence for the hospital's clinical practice (31).

## 7 Discussion

Different arguments could be summarized to explain how antipsychotics can impact pain modulation and management in internal medicine.

### 7.1 Psychopharmacological issues

Many individuals with opioid use suffer from psychiatric disorders like anxiety and depression (47). Antipsychotics, prescribed to manage these psychiatric conditions, can indirectly address the emotional component of pain by treating underlying mood disturbances and improving patients' ability to cope with it (28, 48), influencing cognitive distortions such as catastrophizing.

Antipsychotics often target neurotransmitter systems such as dopamine (49–51), serotonin, and glutamate. These neurotransmitters also play a crucial role in neural pathways of emotional processing. Furthermore, chronic pain can lead to central sensitization, where the nervous system becomes overly responsive to pain signals. Antipsychotics could modulate this process by addressing both pain perception and the central amplification of pain (28, 52). The desensitizing effect is not yet fully understood. Still, it seems to be related to the involvement of many receptors in addition to those for dopamine, which vary according to the antipsychotic being considered: e.g., that mediated by clozapine is hypothesized to be an agonist at the level of μ1-, μ2-, -κ1, -κ3 and α2- opioid receptors, while by olanzapine predominantly at the α2- adrenergic level (53); risperidone especially for μ1-, μ2- and κ1- agonism and to a lesser extent at the level of δ- (54); aripiprazole with partial D2 and 5-HT1A agonism (55).

The co-administration of opioids or nonopioid analgesics with psycholeptics (including antipsychotics) can lead to intricate pharmacodynamic and pharmacokinetic interplays that need to be considered for potential side effects. First-generation antipsychotics have numerous side effects especially extrapyramidal, anticholinergic, and sedative effects (56). Atypical antipsychotics, or second-generation antipsychotics, on the other hand, have a lower affinity for D2 than for 5-HT2A and a concomitant variability of interaction with muscarinic, adrenergic, and histamine receptors possess a pattern with fewer side effects and thus a potential additional treatment to be considered for pain (57). Second-generation antipsychotics are less risky for extrapyramidal effects but are to be cautioned for possible weight gain and metabolic syndrome (56). Third-generation antipsychotics are characterized by better tolerability profiles than their predecessors, especially by less induction of extrapyramidal effects than first-generation or less metabolic impact than second-generation (30). Moreover, the potential risk for additive central nervous system depression raises concerns about sedation with respiratory depression (58) and cognitive impairment (59). It is essential to highlight that the risk of overdose is more significant for more sedative molecules (60).

### 7.2 Clinical implications

Psychiatric comorbidities and the psychological dimension of pain are essential points to consider in optimizing comprehensive pain management, especially in the hospital setting. Interests in implementing evidence-based best practice guidelines cut across healthcare teams (61). The potential antalgic role of antipsychotics, in addition to broadening pharmacologic strategies for pain, would represent a confirmation of the importance of an integrated approach to pain treatment, particularly a consultation-liaison psychiatry and clinical health psychology intervention in internal medicine departments. Such an approach could potentially also promote a reduction in opioid prescribing, scaling back the now well-known problem of the opioid epidemic (7, 8, 62) and overcome some limiting factors for pain treatment such as misperception of pain and communication problems.

### 7.3 Limitations and future perspectives

This study's protocol is subject to limitations inherent to its observational retrospective and longitudinal design, including potential selection bias, missing data, and unmeasured confounding (45, 63). To compensate for those risks, the present protocol plans to use EHR data, which furnish highly reliable data with minor missing information and will control a comprehensive number of clinical variables. To reduce selection bias, exclusion criteria are minimized, and the multisite nature of the Ente Ospedaliero Cantonale Internal Medicine Department will allow for control of specific hospital-related confounders. This study's EHR database does not allow the screening of diagnoses requiring antipsychotic treatment or pain-related diagnoses requiring opioid analgesics treatment, and this study protocol may be subject to indication bias. Our database lacks information on the drug's dosage, the days of treatment supply, and the drug's side effects or adverse events related to drug prescription. Moreover, in-hospital laboratory analysis data are not available in this study EHR database. All this information should be integrated into future studies. EHR data contain sensitive patient information, and strict privacy and security measures must be in place to protect patient confidentiality in future similar studies. Like other observational designs, cross-sequential studies cannot establish causality due to the absence of manipulation or control over variables. To improve its generalizability, this protocol might be applied to other medical specialties concerned with acute pain and the risk of chronicization than internal medicine. In future studies, it will be essential to consider pharmacogenetics data for optimizing personalized pain treatment (64).

## 8 Conclusions

This protocol aims to assess if the use of opioid analgesics and antipsychotics significantly improves pain modulation and management compared to the use of opioid analgesics alone. We expect a more significant reduction in in-hospital VAS score and better management in the short and long post-discharge period in the group with opioid analgesics and antipsychotics. If the results confirm our hypothesis, we will provide real-world evidence supporting future research. Understanding the pharmacological mechanisms underlying these interactions is crucial to designing optimal treatment regimens that minimize risks and maximize benefits. Rigorous research in this area can guide clinicians in tailoring medication regimens, selecting appropriate dosages, and closely monitoring patients for adverse effects. The anticipated outcomes could have implications for integrating the understanding of pain biopsychosocial aspects and supporting patient-centered care that addresses the entirety of the pain experience.
